# ADePT (algorithm for decision-making after pilot and feasibility trials): a decision aid for progression from feasibility study to main trial

**DOI:** 10.1186/1745-6215-14-S1-O19

**Published:** 2013-11-29

**Authors:** Carol Bugge, Brian Williams, Suzanne Hagen

**Affiliations:** 1 University of Stirling, Stirling, UK; 2 Nursing, Midwifery and Allied Health Professions Research Unit, University of Stirling, UK; 3 Nursing, Midwifery and Allied Health Professions Research Unit, Glasgow Caledonian University, UK

## Background

Complex intervention guidance advocates pilot trials and feasibility studies as part of a phased approach to development, testing and evaluation of healthcare interventions. In this paper an example of a feasibility study and pilot trial for a randomised controlled trial (RCT) of pelvic floor muscle training for prolapse is used to explore challenges for progression to main trial.

## Methods

A four-centre feasibility study including pilot trial aiming to randomise 50 women with prolapse of any type or stage and who had had a pessary successfully fitted. We used published methodological categories to classify and analyse the problems that arose in our feasibility study. Subsequently we sought to locate potential solutions that might minimise the trade-off between an explanatory and pragmatic main trial.

## Results

The feasibility study pointed to significant potential problems in relation to participant recruitment, features of the intervention, acceptability of the intervention to participants and outcome measurement. Finding minimal evidence to support our decision-making regarding the transition from feasibility work to a trial, we developed an algorithm (ADePT; **A**lgorithm for **D**ecision-making after **P**ilot and feasibility **T**rials) which we subsequently used as a guide. The algorithm sought to: 1) encourage systematic identification and appraisal of problems and potential solutions; 2) improve transparency of decision-making processes; and 3) reveal tensions that exist between choices which lead to a pragmatic versus explanatory trial.

## Conclusions

We have developed a decision-support tool that may aid future researchers to identify the most appropriate solutions to problems identified within pilot and feasibility studies.

